# Defect-engineered erbium iron garnet *via* high-pressure torsion for enhanced magnetic hyperthermia

**DOI:** 10.1039/d6ra07650e

**Published:** 2026-11-12

**Authors:** Oleg Polozhentsev, David E. Arthur, Zhengyou Li, Kamaludin Abdulvakhidov, Nutan Shukla, Alexander V. Soldatov

**Affiliations:** a The Smart Materials Research Institute, Southern Federal University Rostov-on-Don Russia artur@sfedu.ru; b Department of Pure and Applied Chemistry, University of Maiduguri Nigeria

## Abstract

High-Pressure Torsion (HPT) is a promising severe plastic deformation technique for tailoring the functional properties of magnetic materials. In this study, we investigated the impact of HPT on the magnetic and hyperthermia properties of erbium iron garnet (Er_3_Fe_5_O_12_) particles synthesized *via* a sol–gel auto-combustion method. HPT processing induced significant grain refinement, reducing particles from micron-scale (∼2.3 µm) to the submicron/nanoscale (∼170–550 nm mean size), with ultra-fine fractions as small as 40 nm. While the saturation magnetization (M_S_) decreased due to surface disorder, the Specific Absorption Rate (SAR) improved from 2.8 W g^−1^ (as-synthesized) to 4.396 W g^−1^ (processed at 240 MPa). This enhancement is attributed to a substantial increase in the effective magnetic anisotropy (*K*_eff_) arising from HPT-induced lattice microstrain and defect accumulation, which promote hysteresis-loss-dominated heating under an alternating magnetic field. These results demonstrate that high-pressure torsion is an effective post-synthesis strategy for activating weak ferrimagnetic garnets for magnetic fluid hyperthermia applications, offering a defect-driven pathway to expand the design space of hyperthermia materials beyond conventional iron oxides.

## Abstract 2

High-Pressure Torsion (HPT) is a promising severe plastic deformation technique for tailoring the functional properties of magnetic materials.

## Graphical abstract

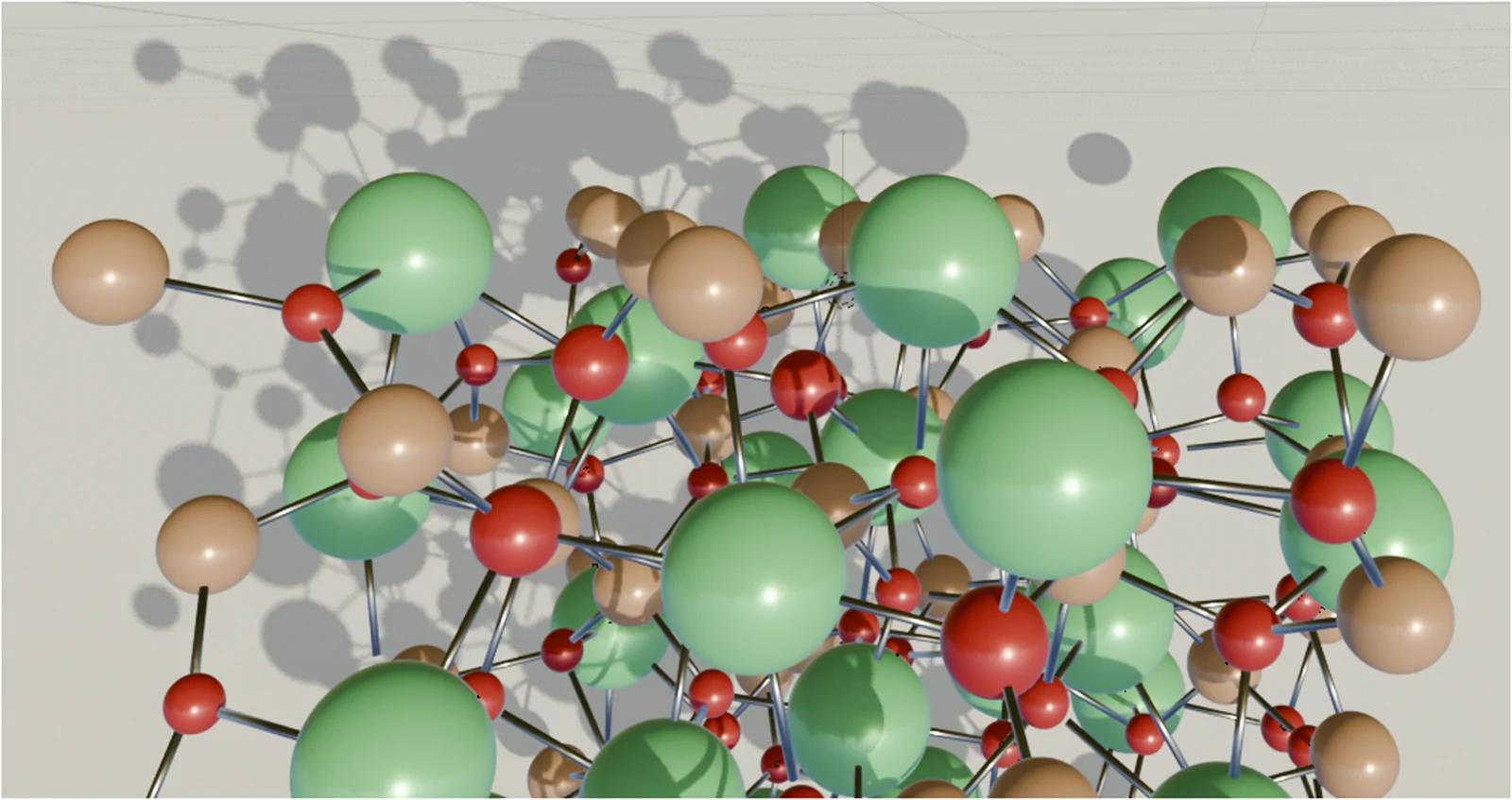

## 1. Introduction

Magnetic fluid hyperthermia (MFH) has emerged as a promising minimally invasive therapy that converts electromagnetic energy into localized heat *via* magnetic nanoparticles under an alternating magnetic field.^1–5^ To date, most MFH research has focused on iron oxide-based nanomaterials, optimizing their heating efficiency through careful control of particle size, shape, surface functionalization, and composition.^5–8^ These strategies have led to significant progress but remain largely confined to materials with inherently high saturation magnetization and well-defined superparamagnetic behavior.

An alternative paradigm is defect-driven MFH, wherein heating efficiency is governed not by magnetization magnitude alone but by defect-induced modifications in magnetic anisotropy and the associated hysteresis losses. In principle, even magnetically “weak” ferrimagnets and complex oxides—traditionally viewed as unsuitable for hyperthermia—could become viable heat mediators if their magnetic anisotropy and coercivity are enhanced through controlled defect engineering.^9,10^ Recent studies have indeed shown that defect-rich magnetic nanoparticles can outperform their defect-free counterparts in generating heat,^11^ highlighting a largely untapped approach. However, experimental demonstrations of defect-driven hyperthermia remain scarce, especially for bulk and particulate oxide systems. One challenge has been identifying post-synthesis techniques capable of introducing a high density of beneficial lattice defects in a controlled manner.

Severe plastic deformation (SPD) methods, particularly high-pressure torsion (HPT), offer a unique route to engineer defects beyond the limits of conventional chemical synthesis,^12–17^ HPT imposes extreme shear strain under high quasi-hydrostatic pressure and produces ultrafine grain sizes together with a high density of dislocations, lattice microstrain, non-equilibrium grain boundaries, and locally broken symmetries, without necessarily inducing amorphization or phase decomposition. The technique has been widely used to tailor the mechanical and functional properties of metals and ceramics,^13,14,18^ its potential as a post-synthesis activation tool for MFH, however, remains largely unexplored. Because the magnetocrystalline anisotropy is highly sensitive to the local lattice environment, the defect structures introduced by HPT can modify the magnetic anisotropy through enhanced spin–orbit coupling and distorted exchange pathways. Such perturbations can raise the coercivity and hysteresis losses even in materials with only moderate magnetization, shifting the optimization strategy for MFH from maximizing the magnetization toward engineering the anisotropy.^18^ Recent reports on doped ferrites support this concept: introducing controlled structural disorder or substitutional defects (*e.g.*, Co or Mn dopants) can double the effective magnetic anisotropy and yield higher hysteresis heating outputs, whereas defect-free analogues rely mainly on Néel or Brownian relaxation.^19–21^

Erbium iron garnet (Er_3_Fe_5_O_12_, ErIG) is a suitable model system for investigating defect-driven hyperthermia. This rare-earth ferrimagnetic garnet possesses a complex magnetic sublattice structure and retains magnetic order up to a high Néel temperature of ≈560 K,^22,23^ yet in conventional microcrystalline form it shows negligible heating under an alternating magnetic field owing to its modest net magnetization. Its magnetic properties are, however, extremely sensitive to structural imperfections such as cation disorder, strain, and other lattice defects,^24^ which makes ErIG an ideal candidate for activation through defect engineering.

Here, we investigate high-pressure torsion (HPT) as a post-synthesis strategy for modifying the structural, magnetic, and magnetic fluid hyperthermia (MFH) responses of Er_3_Fe_5_O_12_. By combining structural characterization, magnetic measurements, first-order reversal curve analysis, calorimetric heating measurements, and first-principles calculations, we examine processing–property relationships and explore their possible origins. HPT processing is associated with microstructural refinement, changes in magnetic reversal behavior, and increased calorimetric heating despite a reduction in the fitted saturation magnetization.^4,8,25,26^ However, the present evidence does not establish a direct causal relationship between specific HPT-induced defects, effective magnetic anisotropy, and enhanced heating.^22,24,27–33^ The calculations illustrate electronic changes associated with modeled defects rather than identify the defects generated experimentally.

The principal contribution of this study is the investigation of processing-dependent behavior within this garnet system, rather than a demonstration of superior heating efficiency, lower production cost, or biological safety relative to established iron oxide formulations. The heating experiments were conducted in aqueous dispersions; biocompatibility and therapeutic efficacy remain to be evaluated.^34–39^

## 2. Material and methods

Stoichiometric amounts of erbium nitrate hydrate [Er(NO_3_)_3_·6H_2_O] and iron(iii) nitrate nonahydrate [Fe(NO_3_)_3_·9H_2_O] were dissolved in 20 mL of deionized water, and 3 g of citric acid (C_6_H_6_O_7_) was added as the fuel. The mixture was gently heated under stirring to evaporate the water until a gel-like precursor formed through the reaction between the metal nitrates and the organic fuel. The temperature was then increased to initiate the auto-combustion reaction. The resulting ash was collected, pressed into a pellet, and heated at 1200 °C for 3 h. The synthesized ErIG powder was finally milled into a fine powder.

### 2.1. High-pressure torsion processing

The synthesized Er_3_Fe_5_O_12_powder was first milled in an agate mortar for 20 min to obtain a homogeneous, fine powder. The milled powder was then loaded between the Bridgman anvils and confined within a metallic ring positioned between them. HPT processing was carried out at room temperature under an applied pressure of 120–280 MPa (step size 40 MPa) by rotating the sample at a low angular velocity of Ω = 0.3 rad min^−1^ for two turns. Six samples were investigated: the as-synthesized powder (S0) and five HPT-processed samples, S1–S5, prepared at 120, 160, 200, 240, and 280 MPa, respectively.

### 2.2. Characterization techniques

The phase composition and structure of the Er_3_Fe_5_O_12_powders were examined using a Bruker D2 Phaser powder diffractometer (30 kV, 10 mA) equipped with a LYNXEYE (1D) detector, using Cu Kα radiation (*λ* = 1.5406 Å) at room temperature. Diffraction data were collected over the 2θ range of 10–80° with a scanning speed of 0.01°/s and a step time of 0.2 s. Scanning electron microscopy (SEM) images were acquired with a field-emission Supra 25 microscope (Carl Zeiss AG, Germany). Magnetization curves *M*(*H*), coercivity (*H*_c_), remanent magnetization (*M*_r_), and first-order reversal curves (FORC) were measured using a Lake Shore VSM Model 7404 magnetometer at room temperature (300 K) with a maximum applied magnetic field of 20 kOe.

First-order reversal curves (FORCs) were measured at 300 K on the same VSM. For each curve, the sample was first saturated in a positive field *H*_sat_, the field was reduced to a reversal field *H*_r_, and the magnetisation *M*(*H*, *H*_r_) was then recorded as *H* was increased back to *H*_sat_. A family of [*N*] curves was acquired with reversal fields spaced by [Δ*H*] Oe over the range [−*H*_1_ … + *H*_2_] Oe. The FORC distribution was obtained as the mixed second derivative.^40,41^
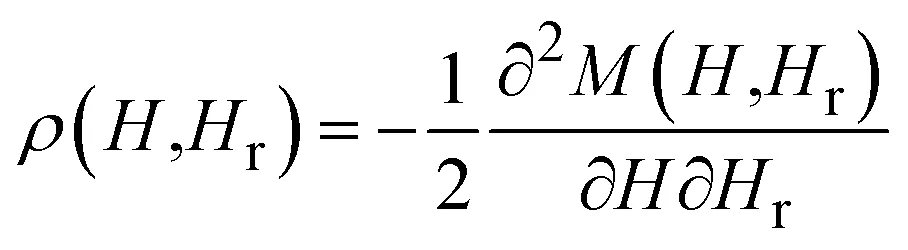
Evaluated by local second-order polynomial fitting with a smoothing factor SF = 10,^41,42^ and plotted in the rotated coordinates
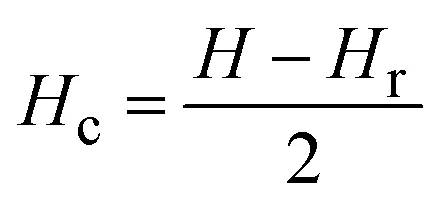

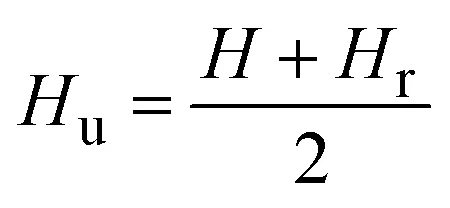
where *H*_c_ is the local switching (coercive) field and *H*_u_ is the local interaction (bias) field.^41,43^ The FORC method originates in the Preisach–Néel description of hysteresis, in which *ρ* is proportional to the Preisach weight function of elementary square hysterons.^40^ This identification is exact only for systems that obey the Mayergoyz wiping-out and congruency properties; for strongly interacting or multidomain assemblies the measured density contains additional features that are not a unique distribution of independent switching units.^44^ The distributions are therefore interpreted here qualitatively, as fingerprints of the switching-field and interaction-field spreads.

Calorimetric magnetic hyperthermia studies were conducted by measuring the time-dependent temperature rise of aqueous dispersions of the ErIG powders using a magnetic hyperthermia setup (Local Hyper Therm Lab Unit TOR04/16, Nanomaterials, Russia). The setup provided alternating magnetic field (AMF) amplitudes up to 20 mT in 2 mT steps at a fixed frequency of 218.9 kHz. The powders were dispersed in an aqueous ethylene glycol (EG) solution; ultrasonic treatment was applied to achieve a uniform dispersion and to increase the settling time of the suspension, allowing more accurate measurements. Dispersions with a particle concentration of 30 mg mL^−1^ were prepared for all measurements. Each dispersion (0.33 mL) was placed in a tube, and the temperature was recorded with a fiber-optic probe inserted at the top of the tube during exposure to the AMF for 30 min. All measurements were repeated several times, and the samples were cooled to room temperature between measurements.

First-principles calculations were performed with the Vienna *ab initio* Simulation Package (VASP 5.3) using the projector-augmented wave (PAW) method and the Perdew–Burke–Ernzerhof (PBE) exchange–correlation functional. The initial crystal structure (POSCAR), INCAR, and *K*-point files were taken from the Materials Project database (https://materialsproject.org/). Supercells containing different point defects—an Er vacancy, an Fe vacancy, and an Er–Fe antisite—were constructed from the pristine structure, and the charge-density distribution of each configuration was calculated and compared with that of the pristine lattice.

### 2.3. Magnetic fluid hyperthermia characteristics

The heating efficiency of MNPs is typically quantified in terms of the specific absorption rate (SAR, W g^−1^ of particles), which can be expressed as:^45^1
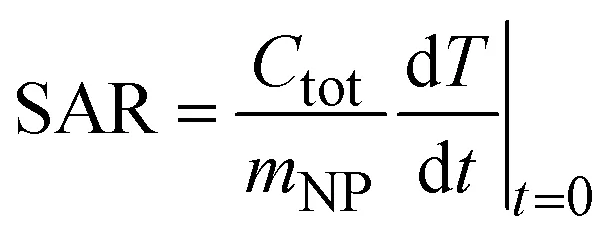
where *C*_tot_ is the total capacity of the nanoparticle dispersion (J K^−1^), *m*_NP_ is the mass of the magnetic nanparticle (g), and d*T*/d*t*|_*t*=0_ is the initial slope of the temperature-time curve (°C/s).

The total heat capacity of dispersion *C*_tot_ of the sample can be determined as:^46^

2 *C*_tot_ = *C*_p,d_*m*_d_ + *C*_p,NP_*m*_NP_

where *m*_d_ and *m*_NP_ are the masses of the dispersion medium of the particles, respectively.*C*_p,NP_ and *C*_p,d_ are the mass-specific heat capacities of the dispersion medium and nanoparticles.^47^

In the present word, the nanoparticles were dispersed in water, because the mass of nanoparticles was much smaller than the mass of the water, the nanoparticles contribution to the total heat capacity was considered negligible:

3 *C*_p,NP_*m*_NP_ ≪ *C*_p,water_*m*_d_

Therefore, the total heat capacity of the dispersion was approximated by the heat capacity of water:

4 *C*_tot_ = *C*_p,water_*m*_d_

where *C*_p,water_ = 4.18 J (g^−1^ °C). The SAR was calculated by:5
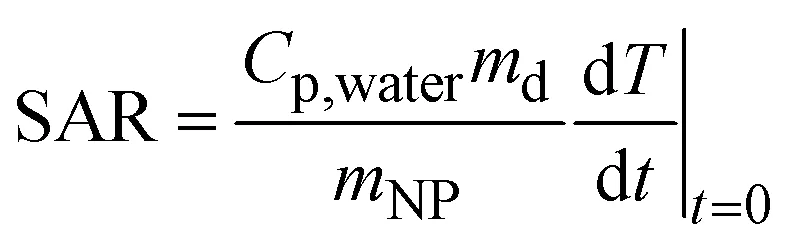


The initial heating rate was determined by fitting the experimental temperature–time data to an exponential function:^48^6
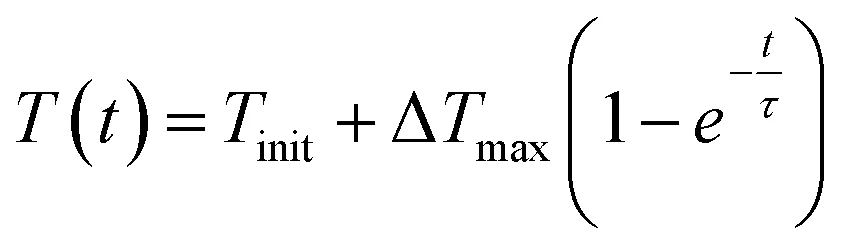
where *T*_init_ is the initial temperature, Δ*T*_max_ is the maximun temperatue increase at long heating times, and *τ* is the heating time constant. So the corresponding initial slope is:7
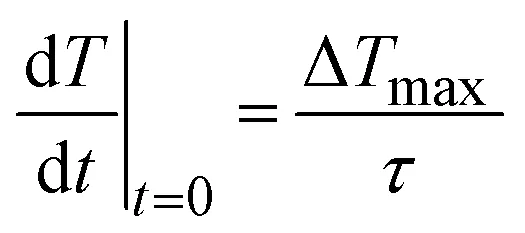


Because the SAR value depends on the frequency *f* and the square amplitude *H*^2^ of the alternating magnetic field, comparisons between different measurements conducted under different conditions should be made cautiously.^49^ To solve this problem, the intrinsic loss power (ILP) is calculated by normalizing the SAR to the AMF strength and frequency:^1,2^8
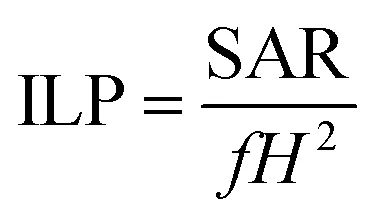


Typically, for single-domain and superparamagnetic MNPs, the ILP value ranges from 0.1 to 1.0 n Hm^2^ kg^−1^.^3–7^

## 3. Results and discussion

### 3.1. X-ray diffraction (XRD) analysis

The X-ray diffraction patterns shown in Fig. 1 display the crystallographic features of the Er_3_Fe_5_O_12_ samples subjected to varying high-pressure torsion (HPT) at pressures ranging from 0 to 280 MPa. The diffraction peaks of all samples were refined using FullProf software with standard references (PDF: #00-023-0240, space group *Ia*3̄*d* (No. 230)), confirming erbium iron garnet as the dominant crystalline phase.^22,24,50^

**Fig. 1:**
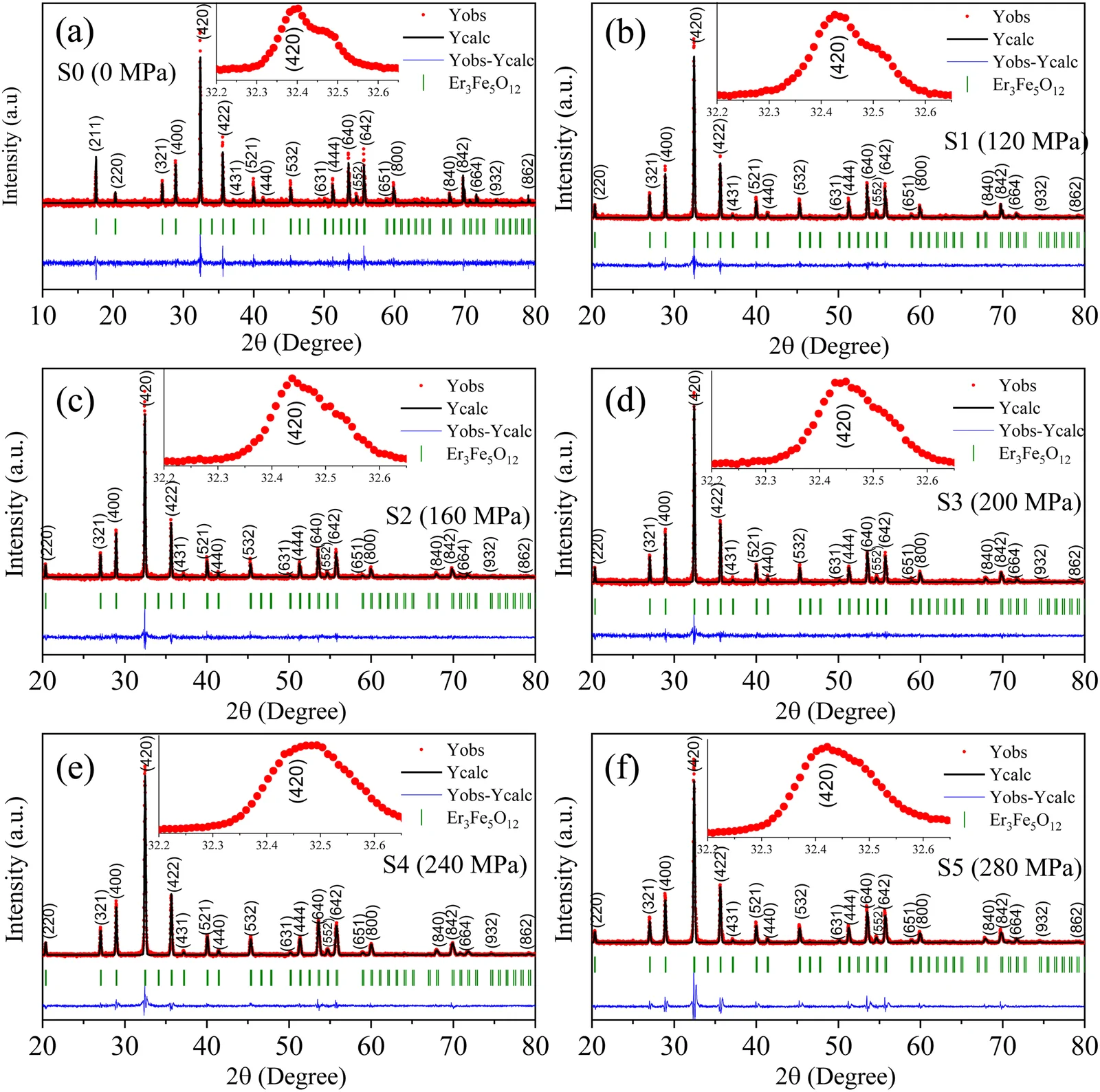
X-ray diffraction (XRD) patterns of Er_3_Fe_5_O_12_ samples subjected to various high-pressure torsion (HPT) conditions: (a) S0 (0 MPa), (b) S1 (120 MPa), (c) S2 (160 MPa), (d) S3 (200 MPa), (e) S4 (240 MPa), and (f) S5 (280 MPa).

The agreement between the observed and calculated patterns supports Er_3_Fe_5_O_12_ as the single phase, which can be detected by XRD throughout the investigated pressure range. Table 1 summarizes the refined structural parameters, characteristics of the (420) reflection, and estimated size of crystallites.

**Table 1:** Crystallographic parameters of Er_3_Fe_5_O_12_

	S0 (0 MPa)	S1 (120)	S2 (160)	S3 (200)	S4 (240)	S5 (280)
*a* = *b* = *c*, Å	12.34956	12.33962	12.33252	12.33415	12.32556	12.33885
*α* = *β*= *γ*,°	90	90	90	90	90	90
FWHM (420)	0.104861	0.112591	0.115295	0.117032	0.170459	0.176914
2*θ*,° (420)	32.394	32.421	32.440	32.436	32.459	32.423
Size, nm	82.46	76.81	75.01	73.89	50.74	48.88
Bragg *R*-factor	10.67	8.855	9.453	8.005	8.024	10.90
RF-factor	11.84	9.883	9.788	8.669	8.500	9.650

The lattice parameter exhibits a slight and monotonous variation with pressure. It decreases from 12.34956 Å for as-synthesized sample S0 to 12.33252 Å for S2, slightly increases to 12.33415 Å for S3, and reaches the lowest value of 12.32556 Å for S4. At 280 MPa it increases to 12.33885 Å for S5 and is less than that of the untreated sample. These changes correspond to the changes in the (420) reflection: lattice contraction is accompanied by a shift to higher diffraction angles, while lattice expansion is accompanied by a shift to lower angles. In particular, the reflection is 2*θ* = 32.394 for S0 and 2*θ* = 32.459 for S4 and 2*θ* = 32.423 for S5. The changes could be associated with deformation-induced lattice distortions, structure defects, point defects, dislocations and residual stresses, or the changes in defect concentration or the type of defects.^18,51^ However, the FWHM of the (420) reflection increases monotonically with pressure, from 0.104861° for S0 to 0.176914° for S5.

The crystallite size is estimated to decrease from 82.46 nm to 48.88 nm. This reduction is nonlinear, as the crystallite size decreases gradually from 76.81 nm for S1 to 75.01 nm for S2 and 73.89 nm for S3, then decreases more significantly to 50.74 nm for S4 and decreases less significantly to 48.88 nm for S5. The most significant change is between 200 and 240 MPa. The results are in agreement with crystallite grinding during HPT, and microstrain associated with deformation-induced defects could also contribute to peak broadening.^52,53^

The trends suggest that HPT facilitates competing processes of fragmentation and defect generation and defect rearrangement/annihilation. These processes may have different effects on the average lattice spacing and local lattice distortions, and thus the pressure dependences of the lattice parameter and diffraction peak width may be different. The lattice expansion observed can be connected to the deformation-induced defects and residual stresses. The non-linear evolution of the peak broadening could be due to a balance between crystallite grinding, accumulated defects, dislocation, point defects, and structural recovery, possibly involving recrystallization. We have found a similar phenomenon in their previous studies.^24,54–59^ The Bragg *R*-factor and (RF)-factor are in the range of 8.005 to 10.90 and 8.500 to 11.84, respectively, defining the agreement between the observed and calculated diffraction intensities.

### 3.2. SEM analysis

The SEM images of the Er_3_Fe_5_O_12_ samples (Fig. 2) show that the particle size and surface morphology are strongly influenced by the HPT processing. In Fig. 2a, the SEM image of the initial powder (S0) shows elongated particles with a grain size ranging from 1 to 6 µm, displaying smooth boundaries and lacking well-defined facets. The average particle size is approximately 2.3 µm, with the smallest particles measuring around 750 nm. In Fig. 2b, the powder sample subjected to a pressure of 120 MPa shows a slight reduction in particle size, with an increased presence of smaller particles that have non-isometric shapes.

**Fig. 2:**
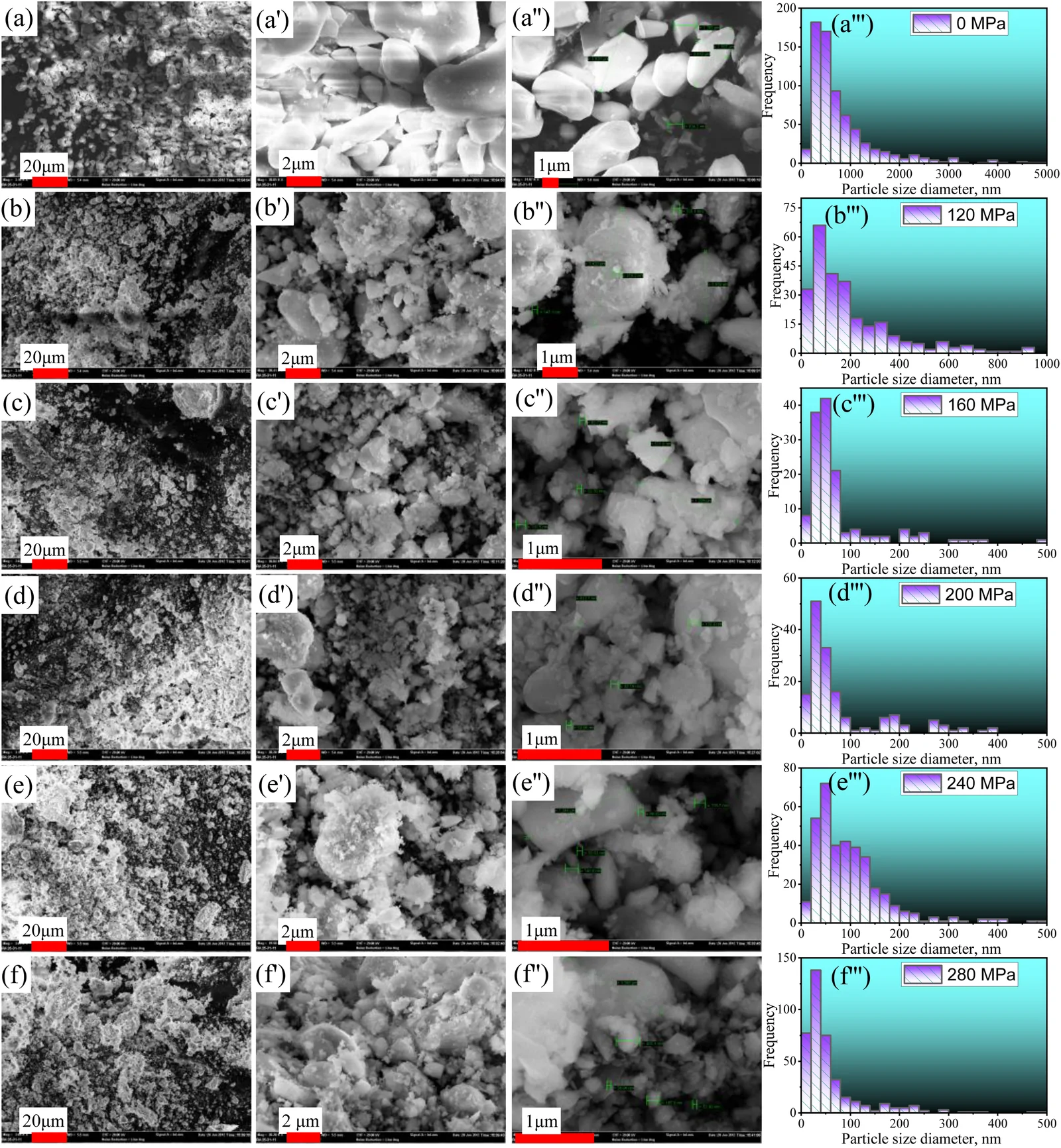
SEM images of Er_3_Fe_5_O_12_ samples subjected to different high-pressure torsion (HPT) conditions, showing the effect of pressure on the particle morphology and size distribution. The images (a)–(f) represent various stages of HPT with corresponding particle sizes at pressures of 0, 120, 160, 200, 240, and 280 MPa, as labeled in the figure. The particle size distributions (a‴)–(f‴) are provided on the right, illustrating the shift in particle size as a function of the applied pressure. As pressure increases, the particle size decreases, reflecting the influence of HPT on the material's microstructure.

As the pressure increases in subsequent samples (Fig. 2c–f), the particle size decreases further. The maximum particle size does not exceed 4.5 µm, and the smallest particles reach approximately 40 nm. The median particle size is reduced to around 170 nm. This morphological evolution is consistent with the combined effects of compression and torsional shear during HPT. Anvil rotation promotes mechanical deformation and interparticle grinding, facilitating fragmentation and the formation of nanosized particles.^33^ Fine fragments may subsequently accumulate on the surfaces of larger particles, producing a coating-like appearance. Interestingly, the crystallite size also decreases significantly with HPT processing, and the relationship between applied pressure and crystallite size is monotonic. Specifically, between 120 and 280 MPa, the crystallite size decreases nonlinearly, which may reflect a balance between crystallite fragmentation, the accumulation of dislocations and point defects, and structural recovery, potentially involving partial recrystallization. For instance, the broadening of the (420) peak in the XRD data is similar for samples at 160 MPa (S2) and 240 MPa (S4), with the maximum broadening observed at 280 MPa (S5), which corresponds to the smallest average crystallite size.

The rotation of the anvil during the HPT process intensifies the mechanical activation, leads to the formation of shear deformations, and accumulates structural defects. As a result, the HPT process causes significant grinding of the material, resulting in the formation of a large number of nanosized particles. Table 2 presents the results of particle size calculations obtained from the SEM images using the ImageJ program. This provides quantified information about the particle size distribution in each sample.

**Table 2:** Statistical parameters of the particle size distribution of all samples obtained from SEM images

Sample (HPT parameter)	Min. size, nm	Max. size, nm	Mean size, nm	Median size, nm
S0 (0 MPa)	750	5300	2370	2250
S1 (120 MPa)	65	3860	700	374
S2 (160 MPa)	63	4550	552	258
S3 (200 MPa)	58	3820	530	218
S4 (240 MPa)	43	4380	513	165
S5 (280 MPa)	40	3450	553	177

Fig. 2(a–f) demonstrate the gradual reduction in particle size as the applied pressure increases from 0 MPa to 280 MPa. This behavior is further confirmed by the corresponding particle size distributions Fig. 2(a‴–f‴), where a clear shift towards smaller particle sizes is observed with increasing pressure. At lower pressures, such as 120 MPa, the particles are larger and more irregular, whereas at higher pressures, particularly at 280 MPa, the particles exhibit a much finer and more uniform structure. These results highlight the effectiveness of HPT in controlling the particle size and improving the material's structural characteristics, which is crucial for enhancing the magnetic properties and potential applications of Er_3_Fe_5_O_12_ in various technologies.

### 3.3. Magnetic properties

The magnetization measurements of all Er_3_Fe_5_O_12_ samples were performed to establish a correlation between the parameters of high-pressure torsion (HPT) processing (pressure in MPa, number of turns) and magnetic properties such as coercivity (*H*_c_), remanent magnetization (*M*_r_), saturation magnetization (*M*_s_), and magnetic hyperthermia properties like specific absorption rate (SAR) and intrinsic loss power (ILP). The hysteresis loops measured at room temperature for each sample are shown in Fig. 3a. All the samples demonstrate ferrimagnetic behavior; however, the magnetization does not saturate at the applied magnetic field. This non-saturation behavior is attributed to the paramagnetic contribution of erbium ions, whose magnetization increases linearly with the magnetic field strength. To quantify the magnetic parameters, the high-field magnetization is analyzed using the law approach to the saturation model, with a ferrimagnetic contribution approaching saturation and an additional field-linear term:^60^9
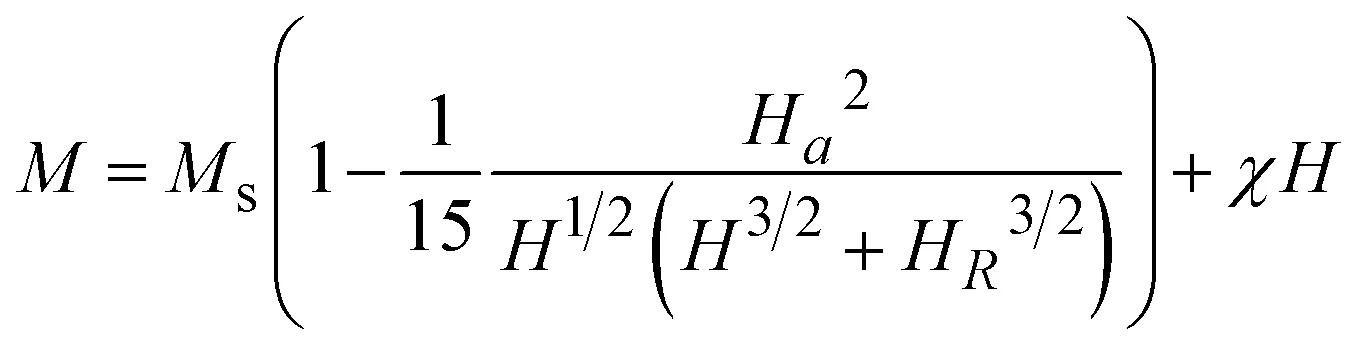
where *M*_s_ is the fitted mass-specific saturation magnetization, *H*_a_ is the local magnetic anisotropy field, *H*_R_ is the exchange field, and *χ*^H^ is the high-field paramagnetic response (emu g^−1^ Oe^−1^).^61–63^ The expression is fitted to positive high-field data. The model parameters and their microscopic interpretation remain dependent on the fitting protocol.

**Fig. 3:**
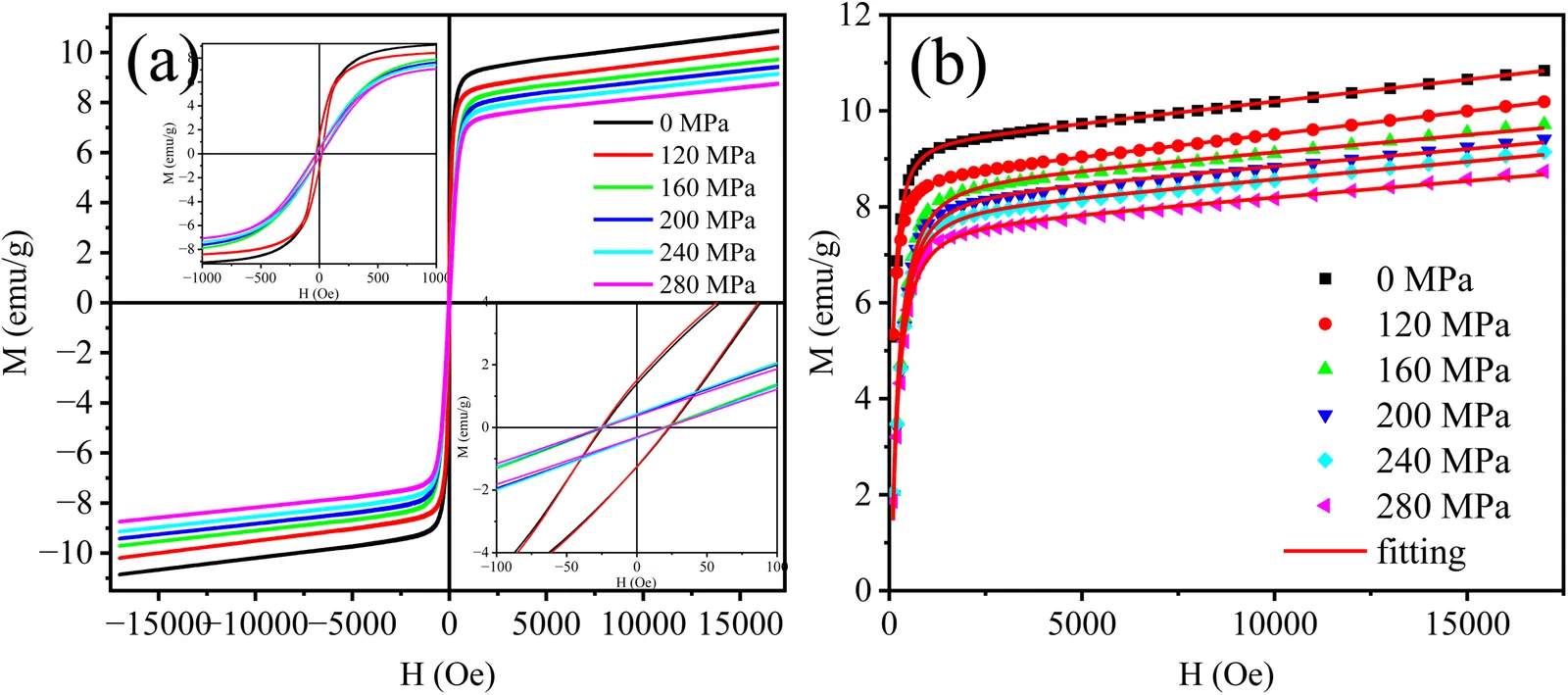
Magnetization (*M*) *versus* applied magnetic field (*H*) curves for Er_3_Fe_5_O_12_ samples subjected to varying high-pressure torsion (HPT) conditions. (a) Hysteresis loops measured at room temperature for samples processed at different pressures (0, 120, 160, 200, 240, and 280 MPa). The inset shows a magnified view of the low-field region, highlighting the differences in coercivity (*H*_c_) across the samples. (b) Fitted data using the law of approach to saturation, showing the magnetization curves fitted to the formula for each pressure condition.

In the CSS convention, the effective anisotropy energy density is

10 *K*_eff_ = *H*_a_*M*_s,vol_/2; *M*_s,vol_ = *ρM*·*M*_s,mass_

Thus, *M*_s_ was expressed per unit volume before *K*_eff_ is reported in erg cm^−3^.

The magnetic characteristics derived from the *M vs. H* curves at room temperature are summarized in Table 3.

**Table 3:** The magnetic characteristics of all samples obtained from *M versus H* curves at room temperature

*P*, MPa	0	120	160	200	240	280
*H*_c_, Oe	23.57	22.92	19.28	20.78	21.07	20.89
*M*_r_, emu g^−1^	1.380	1.496	0.389	0.392	0.428	0.365
2*R*_c,_ nm	82.46	76.81	75.01	73.89	50.74	48.88
*M*_s_, emu g^−1^	9.285	8.570	8.432	8.150	7.857	7.524
*H*_a_, Oe	727.18	706.55	1521.0	1513.0	1415.4	1436.8
*H*_R_, Oe	369	391.48	686.19	681.49	633.29	636.91
*k* × 10^−5^	9.118	9.462	7.145	7.055	7.246	6.783
*K*_eff_ × 10^4^ erg cm^−3^	2.244	2.013	4.264	4.100	3.698	3.594
*M*_r_/*M*_s_	0.149	0.175	0.0461	0.0481	0.0545	0.0485

The saturation magnetization (*M*_S_) tends to decrease with particle size, which is consistent with previous studies.^21,25,26,64^ The as-synthesized erbium iron garnet particles exhibit the highest saturation magnetization value of 9.285 emu g^−1^ (Table 3). However, the particles subjected to a pressure of 280 MPa have the lowest saturation magnetization value of 7.524 emu g^−1^. Nanoparticles obtained through HPT processing with smaller sizes generally show lower saturation magnetization values compared to the 0 MPa sample. This behavior can be attributed to the presence of defects, microstrains, and other factors that contribute to the magnetization variation. The maximum and minimum saturation magnetizations for the HPT-processed samples are 8.570 and 7.524 emu g^−1^, respectively.

The coercivity (*H*_c_) tends to decrease with increasing pressure for most of the samples, as observed in Fig. 3b and Table 3. The initial particles with a particle size of 1–6 µm exhibit a multi-domain structure, where regions of uniform magnetization are separated by domain walls. In multi-domain particles, decreasing particle size leads to increased coercivity due to the inhibition of domain wall motion by defects and microstrains.^34^ Conversely, in single-domain particles, the opposite behavior is expected, where decreasing size results in decreased coercive force.

### 3.4. First-order reversal curve (FORC) study

To further probe how HPT-induced defects influence magnetization reversal and domain interactions, we performed first-order reversal curve measurements. Fig. 4 presents representative hysteresis loops and FORC distributions for selected samples. The FORC density plots provide a “fingerprint” of the magnetic domain state and interactions: peaks in the FORC diagram correspond to prevalent coercive fields and interaction fields in the material.

**Fig. 4:**
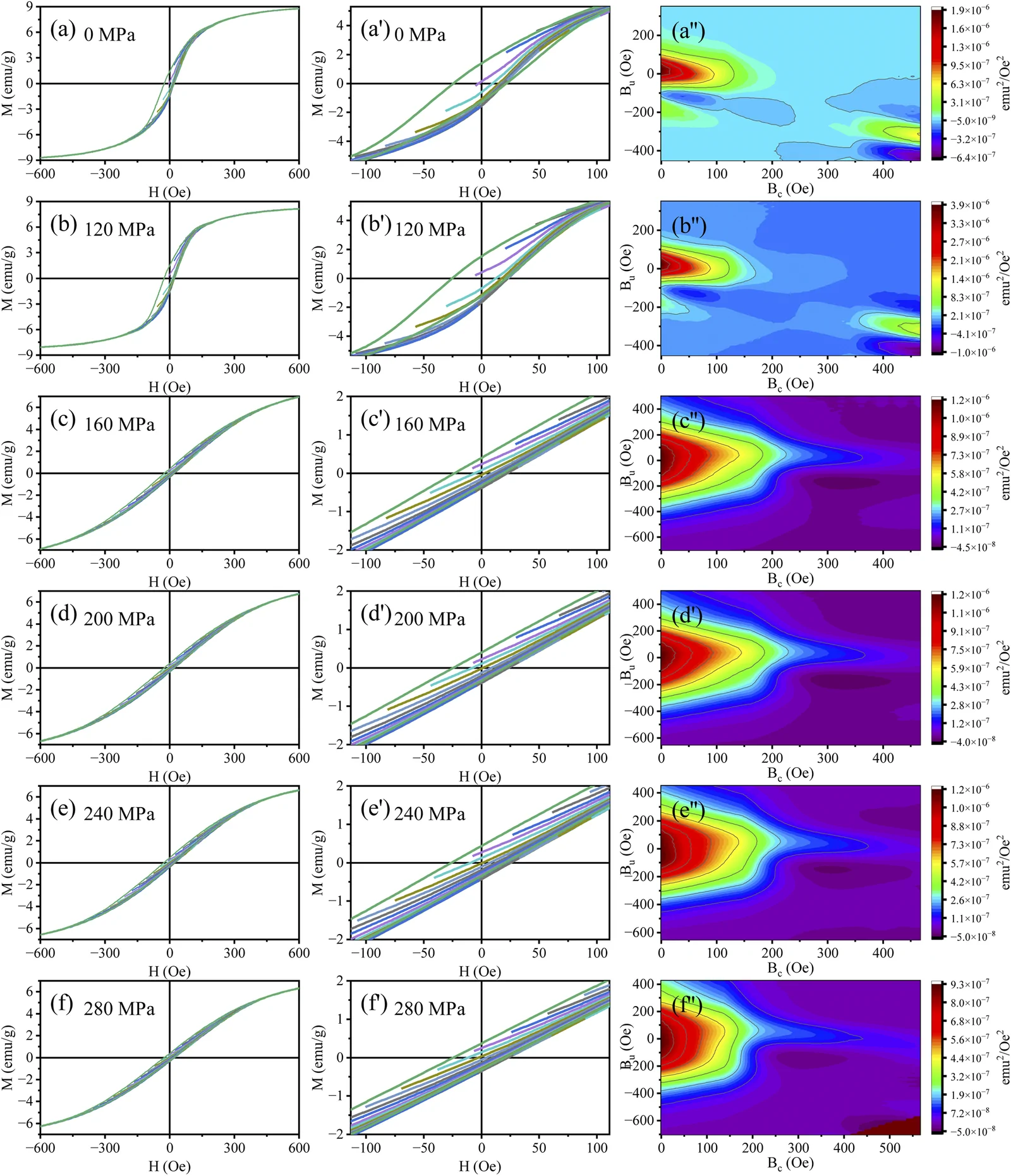
(a–f′) First-order reversal curve (FORC) analysis of Er_3_Fe_5_O_12_ at different HPT states. (a″–f″) The FORC contour plots reveal that undeformed Er_3_Fe_5_O_12_ has broad switching field distributions, whereas HPT-processed samples develop sharper coercive field distributions, indicating more uniform magnetization reversal. Reduced interaction spreads at high pressure reflect weaker dipolar coupling due to the nanoscale dispersion of particles.

The FORC diagrams of S0–S5 are shown in Fig. 4(a″)–(f″) for the field range where the density is non-zero. The as-synthesized sample S0 exhibits a broad distribution along both the *H*_c_ and *H*_u_ axes (Fig. 4a″) which is consistent with a wide distribution of switching fields and strong interaction fields in a polydisperse, mainly multidomain powder.^41,43^ As HPT pressure increases the distribution increase in the *H*_u_ and its maximum moves to higher field in *H*_c_ for S4 and S5 (Fig. 4e″ and f″), with a smaller spread of the interaction field and a more concentrated distribution of the switching field. The FORC measurements are quasi-static and do not alone determine the dynamic loop area at the hyperthermia frequency; their relationship to the SAR values in Section 3.5 is discussed.

The undeformed Er_3_Fe_5_O_12_ (0 MPa) exhibits a broad, diffuse FORC distribution, indicating a wide spread of switching fields and significant magnetostatic/interparticle interactions. This is expected for the polydisperse, multi-domain particles in S0, where domain wall motion and partial reversals occur over a broad field range. In contrast, the high-pressure samples (*e.g.* 240 MPa and 280 MPa) show FORC distributions that are much sharper and more localized. The FORC contours for S4 and S5 concentrate around a relatively well-defined coercivity, with reduced “smeary” background, suggesting a more uniform switching behavior and weaker irreversible interactions.

The FORC diagrams in Fig. 4 illustrate the differences in the magnetization reversal response of the untreated and HPT-processed samples. FORC distributions give information on switching behavior and magnetic interactions. Nonetheless, the features of a FORC distribution can not always be uniquely attributed to individual reversal mechanisms, especially in interacting particle systems. In the present samples, particle refinement and structural changes happen simultaneously during HPT, thus preventing the separation of their individual contributions to the FORC response. We therefore do not consider these plots as evidence for a transition to single-domain behavior or a reduction of interparticle coupling. The primary finding is that the measured reversal response of Er3Fe5O12 is altered by HPT. It is still unknown if these changes can explain the increased SAR and the heating performance is measured separately using the calorimetric measurements in Section 3.5.

### 3.5. Magnetic hyperthermia performance

Calorimetric magnetic hyperthermia studies were conducted on Er_3_Fe_5_O_12_ samples by measuring the time-dependent temperature rise of aqueous solutions containing the powders under exposure to a high-frequency alternating magnetic field (AMF) with different amplitudes (2–20 mT, with a step of 2 mT). Each sample was used at a concentration of 30 mg mL^−1^, which is higher than the typical concentration range (1–3 mg mL^−1^) used in magnetic nanoparticle hyperthermia applications.^2,3,5^ The higher concentration was selected to allow for a qualitative analysis of the magnetic hyperthermia characteristics of the Er_3_Fe_5_O_12_ particles, which generate heat weakly in an external AMF due to their relatively low saturation magnetization.

High-frequency AMF can induce heating in magnetic materials through four independent mechanisms: eddy currents (for particles >1 µm), hysteresis loss (in multi-domain particles with sizes from ∼70–100 nm to 1 µm), and Néel and Brownian relaxations (for single-domain and superparamagnetic nanoparticles).^1,6^ However, eddy currents are significant in magnetic materials of centimeter sizes or greater and therefore in our case, this mechanism can be discarded due to the micron size of as-synthesized ErIG particles. In most cases, the main heating mechanism for magnetic particles of submicron sizes and less with multi-magnetic domains is the hysteresis loss. In this case, heat is generated by thermal energy dissipated through the magnetic domain reversal process that occurs in the ferro- or ferrimagnetic materials. The volumetric power dissipation is proportional to the area of the hysteresis loop and the frequency of the alternating magnetic field. Néel and Brownian relaxations mainly occur in nano-sized particles with single-domain configuration and involve the rapid rotation of magnetic moments within the domain (Néel relaxation) and physical rotation of nanoparticles within the fluid (Brownian relaxation). The main parameters that determine the non-zero area of the hysteresis loop are the saturation magnetization *M*_S_, remanent magnetization *M*_r_ and coercivity *H*_C_, the latter parameter strongly depends on the size of the magnetic core of the particles. The maximum *H*_C_ value of the magnetic material is observed in particles in the region of a single-domain to a multi-domain structure transition, *i.e.* within the size of a single magnetic domain.^1,6^ Therefore, an increase in hyperthermia characteristics can be possible observed with a decrease in the particle size due to the HPT processing.

Fig. 5a shows the time-dependent temperature curves of the aqueous solution for each sample during a 30-minute exposure to an AMF with an amplitude of 20 mT and a frequency of 218.9 kHz. To quantitatively evaluate the heat efficiency, the specific absorption rate (SAR) was calculated, which represents the energy converted into heat per time and mass. The experimental data were fitted with an exponential function *T*(*t*) using formula (3), allowing for the calculation of magnetic hyperthermia characteristics, such as SAR and intrinsic loss power (ILP), using formulas (1) and (4), respectively. The obtained magnetic hyperthermia properties (SAR, ILP) for all samples from the calorimetric magnetic hyperthermia studies are provided in Table 4.

**Fig. 5:**
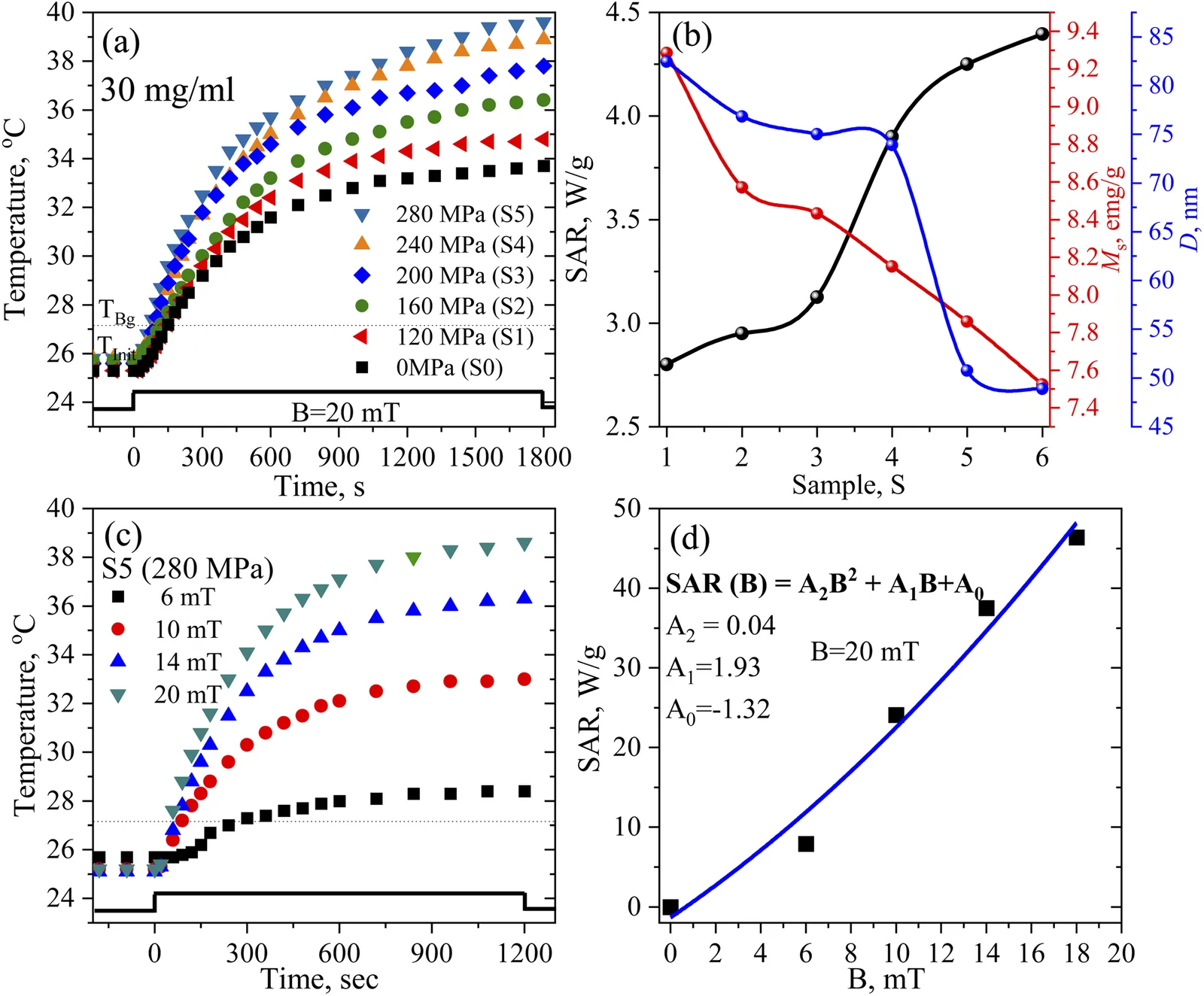
(a) The time-dependent temperature curves of aqueous ferrofluids of the S0–S5 samples during 30 min exposure of AMF with amplitude of 20 mT and a frequency of 218.9 kHz. (b) Relationship between the magnetic hyperthermia properties (SAR), magnetic properties (*H*_C_, *M*_S_) and the parameters of the HPT processing. (c) The time-dependent temperature curves of aqueous ferrofluids of the S5 samples during 20 min exposure of AMF with various amplitudes. (d) SAR of the S5 sample *versus* magnetic field amplitude.

**Table 4:** The magnetic hyperthermia properties for all samples obtained from calorimetric magnetic hyperthermia studies^a^

Sample	Δ*T*_max_ (°C)	*τ* (sec)	ν = Δ*T*_max_/*τ* = (d*T*/d*t*)|*t* = 0, (°C/sec)	SAR (W g^−1^)	ILP (nH m^2^ kg^−1^)
S0 (0 MPa)	9.1	450.27	0.02	2.8	0.032
S1 (120 MPa)	10.56	467.1	0.0226	3.126	0.036
S2 (160 MPa)	11.74	550.6	0.0213	2.95	0.034
S3 (200 MPa)	14.02	496.95	0.0282	3.9	0.045
S4 (240 MPa)	14.13	444.47	0.0318	4.396	0.05
S5 (280 MPa)	12.55	408.41	0.0307	4.25	0.048

a Where *C* = 1.3827 J K^−1^, *m*_NP_ = 10 mg, *f* = 218900 Hz, *H* = 0.02*T*.

The SAR of the as-synthesized powder (S0 sample) was found to be 2.8 W g^−1^, which is relatively low compared to typical magnetic nanoparticles used in magnetic fluid hyperthermia (MFH) applications. The maximum temperature increase observed in the aqueous solution of the S0 sample (30 mg mL^−1^, 20 mT, 218.9 kHz) with particle sizes ranging from 1 to 6 microns was approximately 34 °C.

However, when the initial powder was subjected to HPT processing, the heating temperature of the treated powders increased. The aqueous solution of the sample subjected to a pressure of 10 T reached a temperature of approximately 37 °C, while the other samples subjected to HPT processing exhibited varying heating temperatures. The SAR values for the treated samples ranged from 2.95 to 4.396 W g^−1^, representing an improvement in hyperthermia characteristics compared to the as-synthesized sample. The HPT processing enhanced the magnetic hyperthermia properties of the samples, making it a potential post-synthesis method for processing ferromagnetic materials to optimize their application in magnetic fluid hyperthermia. The maximum heating temperature of around 40 °C was observed for sample S5 processed at 280 MPa.

In Fig. 5b, the relationship between the magnetic hyperthermia properties (SAR), magnetic properties (*H*_C_, *M*_S_), and the parameters of the HPT processing for all samples is shown. Fig. 5c illustrates the dependence of the heating temperature of the aqueous solution on the exposure time to AMF with different amplitudes (6–20 mT). Fig. 5d demonstrates the observed linear dependence of SAR values on the amplitude of the AMF. However, further research is necessary to explore the application of HPT processing to both ferromagnetic multi-domain particles and single-domain or superparamagnetic nanoparticles (Table 5).

**Table 5:** Compares the heating performance of the present Er_3_Fe_5_O_12_ samples with reported iron garnets and a conventional magnetite benchmark

Material and preparation	Size and measurement basis	Dispersion medium; concentration	Frequency (kHz)	Reported field amplitude	SAR (W g^−1^)
Y_3_Fe_5_O_12_, sol–gel synthesis and calcination	Reported particle-size range across the prepared materials: 100–400 nm (ref. 65)	Medium not specified in the accessible abstract; 1 mg mL^−1^	Not verified	Not verified	114.65
Y_3_Fe_5_O_12_, microwave-assisted sol–gel auto-combustion route	XRD crystallite size: 56.45 nm; mean SEM grain size: 1.36 µm (ref. 66)	Hexane; 1 mg mL^−1^	316	Approximately 35.2–35.33 kA m^−4^	237
Y_3_Fe_5_O_12_, microwave-assisted hydrothermal route	XRD crystallite size: 59.95 nm; mean SEM grain size: 2.30 µm (ref. 66)	Hexane; 1 mg mL^−1^	316	Approximately 35.2–35.33 kA m^−4^	214
Fe_3_O_4_, coprecipitation followed by PEG coating, sample S3	Reported average particle/crystallite size: 12.2 nm (ref. 67)	Water; 5 mg mL^−1^	765.85	350 G, equivalent to 35 mT	145.84 (ref. 5)
Fe_3_O_4_, coprecipitation followed by PEG coating, sample S6	Reported average particle/crystallite size: 10.2 nm (ref. 67)	Water; 5 mg mL^−1^	765.85	350 G, equivalent to 35 mT	81.67

### 3.6. Theoretical insights from DFT

To elucidate how atomic-scale defects alter the electronic and magnetic structure of Er_3_Fe_5_O_12_, we carried out density functional theory (DFT) charge-density difference calculations for several modeled defects. First-principles studies of iron garnets have shown that their magnetic exchange interactions and magnetocrystalline anisotropy are highly sensitive to the local electronic structure;^68–70^ the calculations below examine how point defects perturb the charge density and hence these magnetic couplings. Fig. 6 visualizes the charge-density perturbations caused by representative point defects: an erbium vacancy (missing Er ion), an iron vacancy (missing Fe ion), and an Er–Fe antisite swap. In each case, the introduction of the defect leads to a pronounced redistribution of the electron density around the defect site relative to the pristine lattice. For example, removing a central Er ion (Fig. 6a and a′) causes the electron density to contract around the vacancy and the neighboring oxygen/polyhedral units, reflecting the reorganization of the bonding in the absence of the trivalent Er^3+^cation. Similarly, an Fe vacancy (Fig. 6c and c′) depletes the charge in the vicinity of the missing Fe and causes subtle rearrangements on the adjacent Er–O bonds.^71,72^

**Fig. 6:**
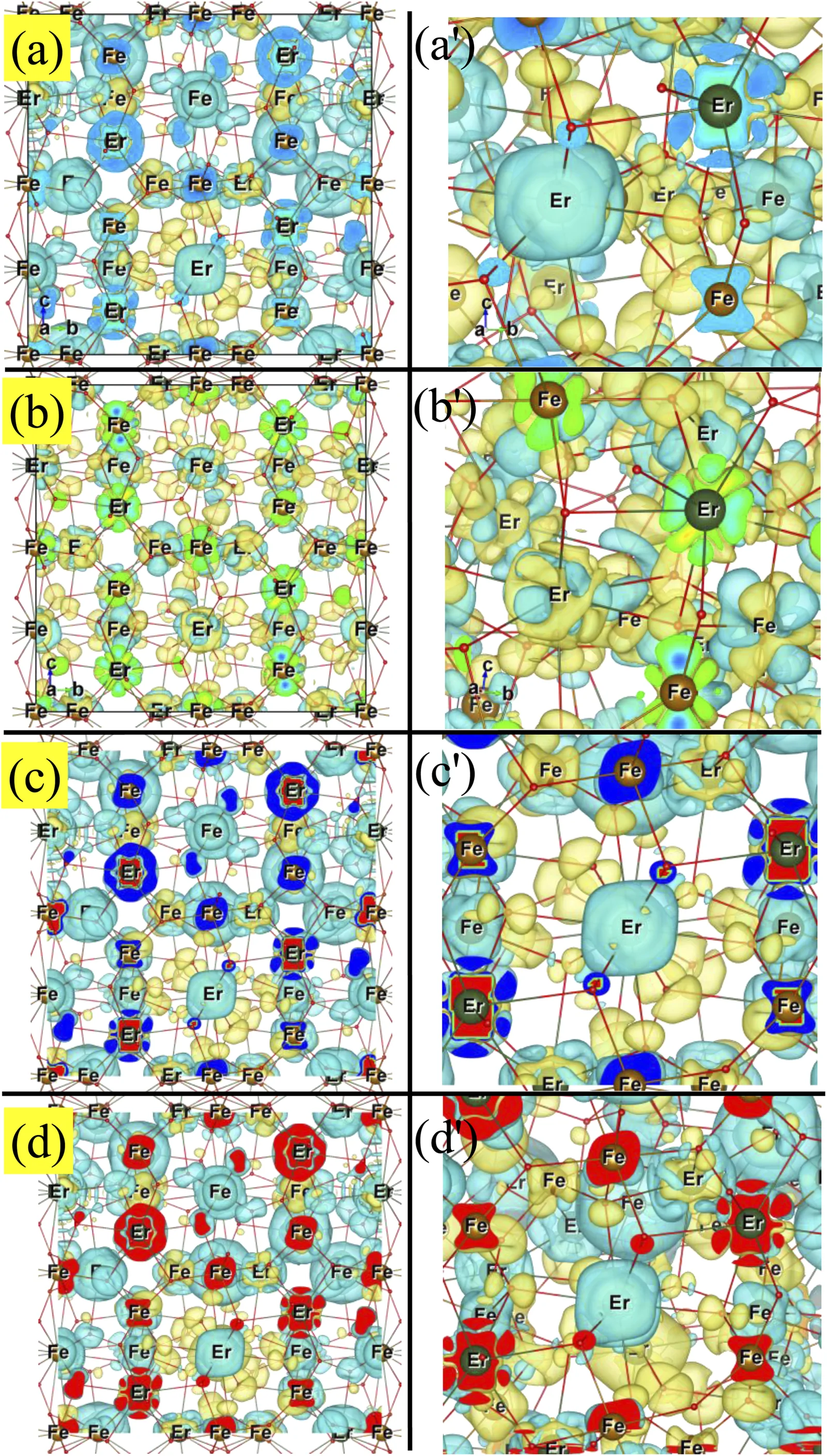
DFT-calculated charge density difference isosurfaces for Er_3_Fe_5_O_12_ with various lattice defects (yellow/red lobes indicate regions of increased/decreased electron density compared to the pristine crystal). (a and a′) Central Er atom present *vs.* removed (Er vacancy): electron density contracts around the vacancy when Er is removed. (b and b′) With one Er vacancy at the center of the supercell: significant local charge redistribution occurs, primarily near where the Er was removed. (c and c′) With one Fe atom removed (Fe vacancy): charge is depleted at the vacant Fe site and rearranged among neighboring O and Er atoms. (d and d′) Er–Fe antisite swap defect: an Er and Fe exchange positions, causing a complex reconfiguration of charge density in their environs. These simulations illustrate how lattice defects perturb the electronic structure, which in turn can modify magnetic properties (exchange coupling and anisotropy) in Er_3_Fe_5_O_12_.

The Er–Fe antisite swap (Fig. 6d and d′) produces an even more complex pattern of charge redistribution, because the swapped ions disrupt the normal sublattice ordering and create a mixed coordination environment. These charge-difference maps show that each type of defect perturbs the local electron density in a distinct way and can therefore modify the magnetic exchange interactions.^73^ In the garnet structure, the Fe–O–Fe superexchange pathways largely determine the net magnetization and the anisotropy;^74,75^ defects that alter the Fe–O bond lengths or angles, or place unpaired Er 4f moments in unusual sites, shift the balance between the magnetic sublattices and locally change the magnetic energy landscape.^76^

Our DFT results qualitatively confirm that vacancies and antisite defects can locally modify the magnetic anisotropy by breaking the local symmetry and creating uncompensated magnetic moments. Defect engineering is thus an effective route to tune the magnetic energy landscape of Er_3_Fe_5_O_12_, in line with recent first-principles studies of defect-induced magnetism in oxides.^77^ Projected plane-wave bonding analyses such as the crystal orbital Hamilton population (COHP) method implemented in the LOBSTER code have likewise been used to quantify how local charge rearrangements affect bonding and magnetic exchange in complex oxides.^78,79^ The experimentally observed increase in the effective anisotropy of the HPT-processed samples, achieved through a high density of such defects, is well supported by these atomistic insights: HPT introduces a spectrum of point and extended defects, and the DFT analysis shows how these defects redistribute the charge and modify the magnetic interactions. This reinforces our interpretation of the hyperthermia improvement as a defect-driven electronic-structure effect.

To complement the charge-density analysis of the defect cells, the electronic structure of the pristine Er_24_Fe_40_O_96_ supercell was characterized by the orbital-resolved densities of states (DOS) and a projected bonding analysis. Fig. 7 shows the orbital-resolved DOS per atom obtained without and with spin polarization. In the non-spin-polarized case (Fig. 7B), the O 2p states form a band centered ≈3 eV below the Fermi level, separated by a pronounced minimum from the Fe 3d manifold, and the Fermi level falls at the foot of a steeply rising unoccupied Fe 3d feature, where the DOS is reduced to ≈37 states per eV. Upon spin polarization (Fig. 7A), the Fe 3d manifold is exchange-split by ≈ 1.3 eV: the majority-spin states are fully occupied, whereas the minority-spin states cross the Fermi level and yield a metallic-like DOS. The Er-projected states are predominantly of d character and remain largely unoccupied, with their main peak ≈3.7 eV above the Fermi level and only minor s and p contributions in the valence region. The orbital-resolved picture therefore identifies the Fe 3 d–O 2p hybridization as the dominant electronic channel in Er_3_Fe_5_O_12_.

**Fig. 7:**
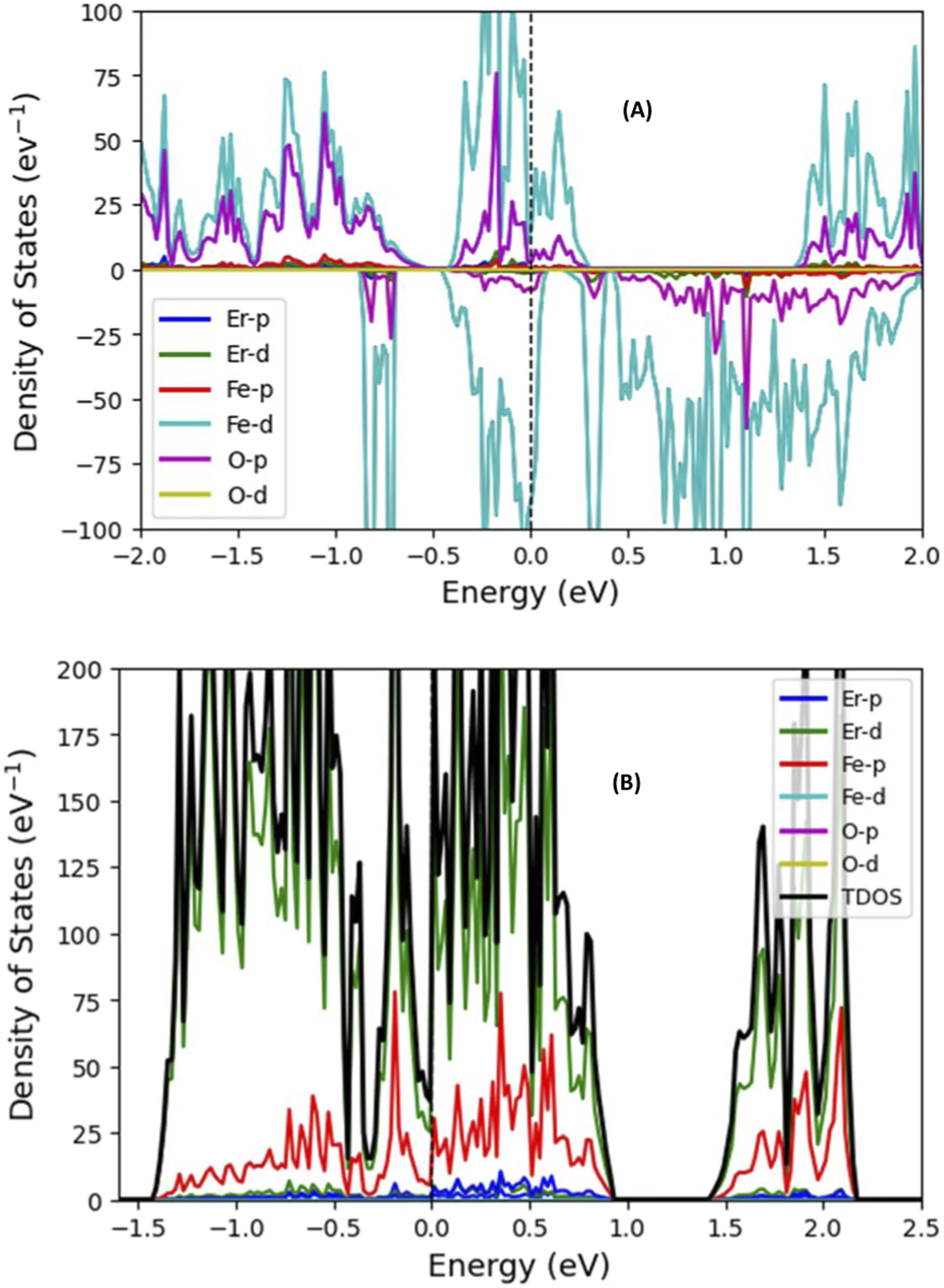
(A) Spin-polarized orbital-resolved DOS and (B) non-spin-polarized orbital-resolved DOS for the total p and d orbitals per atom.

Fig. 8 compares the total DOS with the element-projected contributions of Er, Fe, and O. In both spin treatments, the states at the Fermi level are overwhelmingly of Fe 3d character with a substantial O 2p admixture; the Er contribution is small (≈2–4%), consistent with the mostly unoccupied Er 5d states. In the spin-polarized ground state, the total DOS at the Fermi level reaches ≈128 states per eV and is dominated by minority-spin Fe 3d states (≈2.3 *vs.* 0.4 states per eV per Fe atom). The spin-polarized solution carries a total magnetic moment of 112.34 µB per 160-atom cell (14.04 µB per formula unit) and is stabilized by 20.36 eV per cell (2.55 eV per formula unit) relative to the non-spin-polarized state, confirming that the ferrimagnetic ground state is strongly favored over the nonmagnetic reference.

**Fig. 8:**
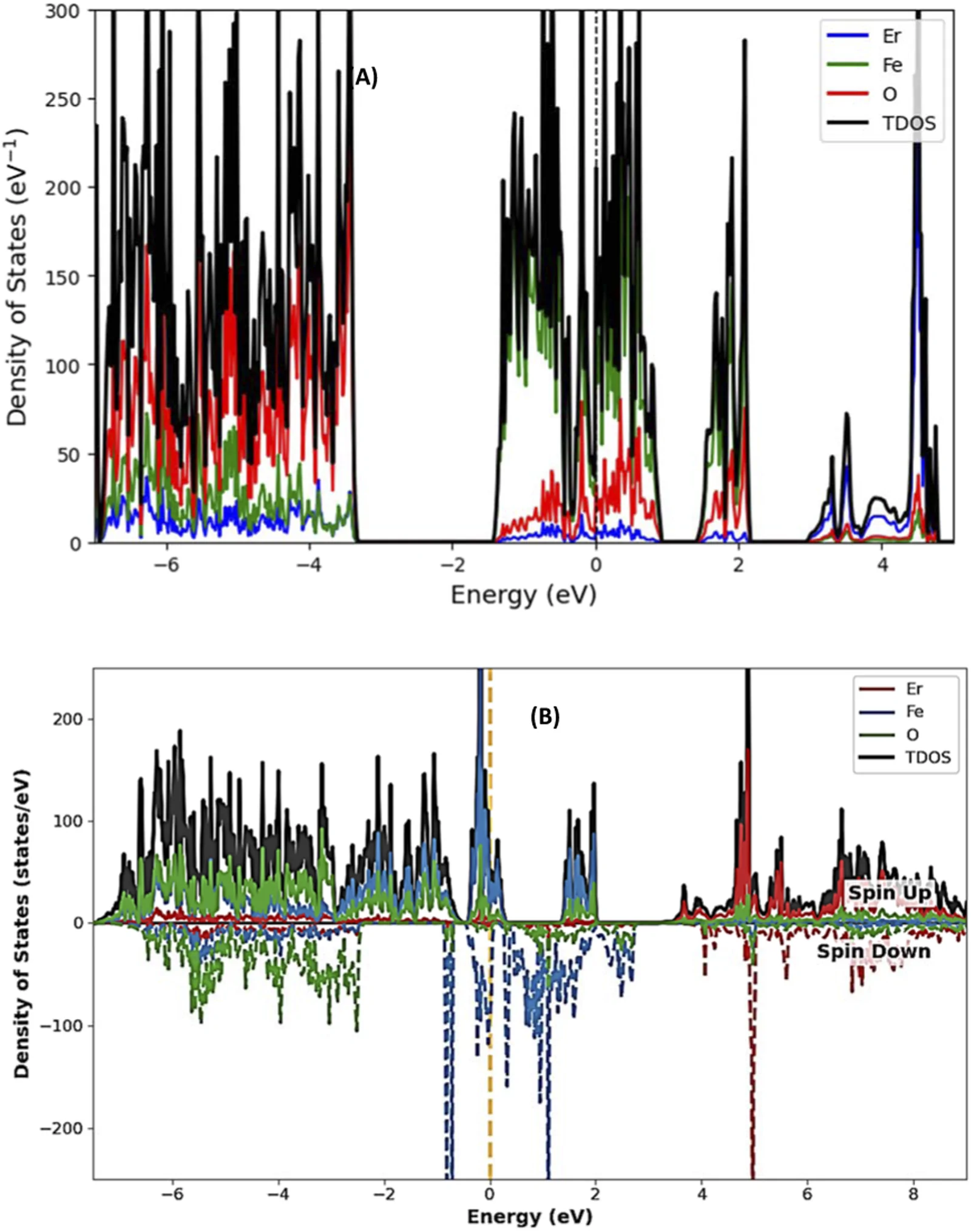
Total DOS and element-projected DOS (per atom) of Er, Fe, and O in Er_3_Fe_5_O_12_ for (A) non-spin polarization and (B) spin polarization effect.

Fig. 9 quantifies the bonding hierarchy of the dominant interatomic interactions through the crystal orbital Hamilton population (COHP) and crystal orbital overlap population (COOP) analyses projected from the plane-wave basis. The integrated values identify the Fe–O bonds (≈1.9 Å) as strongly covalent (ICOHP ≈ −1 eV per pair), whereas the Er–O bonds (≈2.4 Å) are only weakly bonding (ICOHP ≈ −0.3 eV per pair) and the cation–cation contacts (Fe–Fe ≈ 3.4 Å and Er–Fe ≈ 3.1 Å) are essentially nonbonding, with ICOHP magnitudes below ≈0.05 eV per pair. This hierarchy confirms that the magnetic exchange in the garnet lattice is carried by the Fe–O–Fe superexchange pathways^68,69^ and explains why point defects that distort or remove Fe–O bonds (Fig. 6) perturb the exchange couplings and the magnetic anisotropy so effectively. Together with the DOS analysis, these results provide a first-principles basis for the defect-driven enhancement of the effective magnetic anisotropy inferred from the FORC and magnetic hyperthermia measurements.

**Fig. 9:**
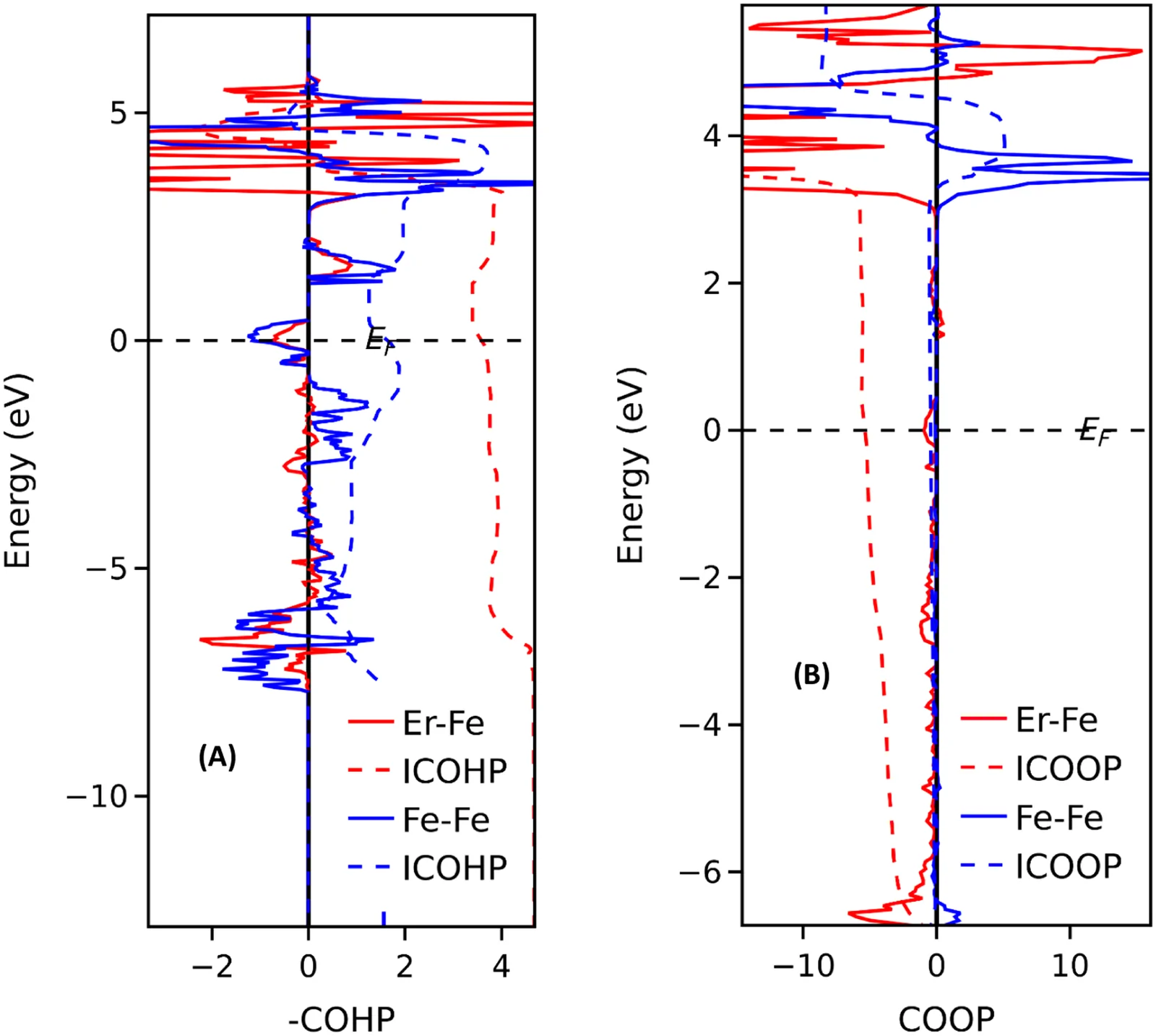
pCOHP and ICOHP curves (A) and (B) COOP and ICOOP curves for the Er–Fe and Fe–Fe interactions.

## 4. Conclusions

In this work, fine-grained erbium iron garnet (Er_3_Fe_5_O_12_) powders were successfully synthesized using a sol gel auto-combustion method and subsequently modified by high-pressure torsion (HPT) under pressures up to 280 MPa. Severe plastic deformation through torsional straining led to pronounced particle fragmentation and microstructural refinement, producing submicron and nanoscale particles with sizes down to ∼40 nm. This structural evolution was accompanied by the introduction of a high density of lattice defects and microstrains, profoundly altering the magnetic response of the material.

HPT processing resulted in a moderate reduction of the saturation magnetization due to surface disorder and defect-induced spin canting, while simultaneously modifying coercivity and expanding the hysteresis loop area. These defect-driven magnetic changes translated directly into improved magnetic hyperthermia performance. In particular, the specific absorption rate increased significantly compared to the as-synthesized material, demonstrating that enhanced effective magnetic anisotropy and hysteresis losses rather than magnetization magnitude alone govern the heating efficiency of Er_3_Fe_5_O_12_ under alternating magnetic fields. The monotonic dependence of magnetic and hyperthermia properties on applied pressure highlights the existence of an optimal defect and strain state, beyond which excessive disorder can partially suppress performance.

These findings establish high-pressure torsion as a powerful post-synthesis strategy for activating weak ferrimagnetic materials for magnetic fluid hyperthermia applications. By enabling controlled defect engineering and microstructural refinement without altering the chemical composition or crystal phase, HPT offers a versatile route to enhance functional magnetic properties in materials traditionally considered unsuitable for hyperthermia. Future work should focus on extending this approach to a broader range of ferrimagnetic systems, as well as systematically exploring the interplay between defect density, particle size regime (multi-domain, single-domain, and superparamagnetic), and alternating-field conditions to fully exploit the potential of severe plastic deformation for next-generation hyperthermia materials.

## Author contributions

Oleg Polozhentsev: conceptualization, data curation, investigation, writing – original draft; David E. Arthur: writing – original draft, review & editing, formal analysis, DFT (COHP, COOP, DOS) computation; Zhengyou Li: writing- review & editing, investigation, software, validation, resources; Kamaludin Abdulvakhidova: resources, supervision; Nutan Shukla: data curation, conceptualization, formal analysis; Alexander V. Soldatov: project administration, funding acquisition.

## Conflicts of interest

The authors declare no conflict of interest.

## Data Availability

The data that support the findings of this study, including X-ray diffraction patterns, electron microscopy images, magnetisation curves, and magnetic hyperthermia measurements, are available from the corresponding author, David E. Arthur, upon reasonable request.
